# Emergency room imaging in pediatric patients with cancer: analysis of the spectrum and frequency of imaging modalities and findings in a tertiary cancer center and their relationship with survival

**DOI:** 10.1186/s40644-021-00421-7

**Published:** 2021-08-28

**Authors:** Sungmin Woo, Abdallah Araji, Mohammad Ali El Amine, Natalie Gangai, Elizabeth Acquafredda, Anita P. Price, Tanya M. Trippett, Hedvig Hricak, Hebert Alberto Vargas, Gerald G. Behr

**Affiliations:** 1 Department of Radiology, Memorial Sloan Kettering Cancer Center, 1275 York Ave, 10065 New York, NY USA; 2 Department of Pediatrics, Memorial Sloan Kettering Cancer Center, New York, NY USA

**Keywords:** Emergency room, Imaging, Pediatric cancer, Admission, Survival

## Abstract

**Background:**

To assess the spectrum and frequency of modalities used for emergency room (ER) imaging and their findings in pediatric cancer patients and assess their relationship with survival.

**Methods:**

Consecutive pediatric cancer patients that underwent imaging during an ER visit at our tertiary cancer center over a 5-year period were retrospectively analyzed. Imaging findings were considered positive when they were relevant to the ER presenting complaint. Imaging positivity was correlated with inpatient admission. Overall survival (OS) was assessed with Kaplan-Meier curves and uni- and multi-variate Cox proportional hazards model was used to identify significant factors associated with OS.

**Results:**

Two hundred sixty-one patients (135 males and 126 females; median age 11 years [interquartile range 5–16 years] with 348 visits and a total of 406 imaging studies were included. Common chief complaints were related to the chest (100 [28.7 %]) and fever (99 [28.4 %]). ER imaging was positive in 207 visits (59.5 %), commonly revealing increased metastases (50 [14.4 %]), pneumonia (47 [13.5 %]), and other lung problems (12 [2.9 %]). Positive ER imaging was associated with inpatient admission (69.3 % [133/192] vs. 40.4 % [63/156], *p* < 0.01). Multivariate survival analysis showed that positive ER imaging (hazard ratio [HR] = 2.35 [95% CI 1.44–3.83, *p* < 0.01), admission (HR = 1.86 [95% CI 1.17–3.00], *p* < 0.01), number of ER visits (HR = 3.08 [95% CI 1.62–5.83], *p* < 0.01 for ≥ 3 visits) were associated with poorer survival.

**Conclusions:**

Imaging was able to delineate the cause for ER visits in children with cancer in over half of the cases. Positive ER imaging was associated with admission and worse survival.

## Background

Despite vast improvements in cancer treatment in recent years, cancer ranks third among the leading causes of death in children and adolescents, accounting for approximately 9 % of all deaths [1]. In addition to requiring specialist treatment, pediatric cancer patients are at risk of complications presenting as oncologic emergencies. Oncologic emergencies can represent a diagnostic challenge, as patients are susceptible to conditions related to their cancer, cancer treatment, but also to general emergency conditions which can occur in any patient. This is further accentuated in pediatric oncologic emergencies, which differ from those found in their adult counterparts due to differences in physiology, type of common cancers, and their treatments [2]. Moreover, young children typically do not verbalize their symptoms accurately, leading to further reliance on imaging for accurate diagnosis. As a result, it is important to understand nuances specific to the pediatric population to facilitate timely recognition and management.

Imaging plays a crucial role in the detection and management of oncologic emergencies in both adult and pediatric cancer patients [3–7]. However, unlike in the adult population where there is a high utilization rate of computed tomography (CT), alternate modalities such as ultrasound (US), radiographs and more recently magnetic resonance imaging (MRI), are favored in the pediatric population when clinically appropriate, in order to reduce potentially harmful long term cumulative effects of radiation exposure [5]. Although many previous review articles have provided excellent overview and illustrations of typical pediatric oncologic emergencies and their imaging findings, it is not well established how imaging is utilized in terms of the spectrum and frequency of imaging modalities, their yield or positivity in identifying the cause of the symptoms, and the relationship to clinical outcomes. Such information would be of incremental value for health care providers caring for pediatric patients with potentially life-threatening emergencies visiting the emergency room (ER).

The purpose of this study was to assess the spectrum and frequency of the modalities used for ER imaging and their findings, and to evaluate their association with clinical outcomes in pediatric patients with cancer treated in the setting of a tertiary cancer center.

## Methods

### Patient selection

**Fig. 1:**
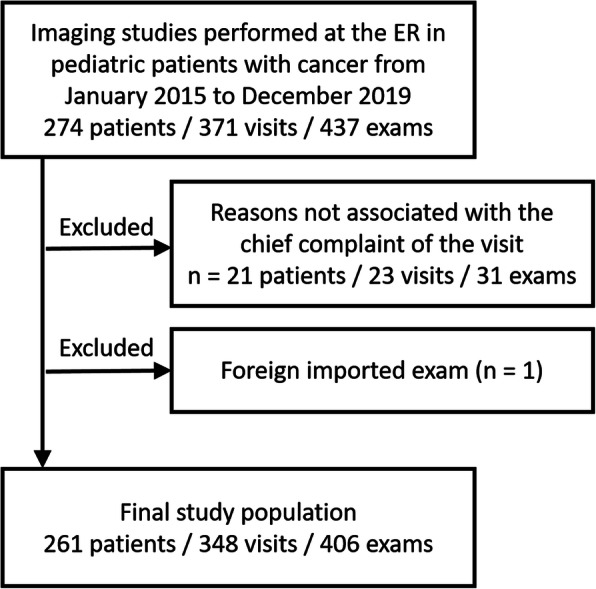
Flowchart showing patient selection process

This study received approval from the institution review board and was compliant with the Health Insurance Portability and Accountability Act. The institutional electronic medical records and PACS database were searched to identify pediatric cancer patients (age ≤ 18 years) who had any type of diagnostic imaging test done as part of the visit to the ER at ***** from January 2015 to December 2019. We initially identified 274 patients / 371 visits / 437 exams. Exclusion criteria were as follows: (1) imported examination from an outside institution (*n* = 1) and (2) imaging performed at the ER but for reasons not associated with chief complaint (e.g., line check after insertion of central venous catheter as part of the care during the ER visit) (*n* = 21 patients / 23 visits / 31 exams). Ultimately, 261 patients with a total of 348 visits / 406 exams were included for the study population (Fig. 1).

### Imaging and clinical characteristics

The spectrum and frequency of the imaging modalities and their imaging findings were collected from the radiology reports. The imaging modalities were categorized as radiographs, CT, MRI, positron emission tomography (PET/CT), other nuclear medicine examinations, and US. Imaging findings were considered positive vs. negative based on whether they were related to the reason for the visit to the ER and the positive findings were classified into the following categories [4]: (1) abscess or complicated fluid, (2) enteritis, colitis, or other bowel inflammation, (3) bowel obstruction, (4) pneumatosis, (5) urinary tract infection, (6) pneumonia, (7) other pulmonary findings (e.g., pneumothorax, pulmonary embolism), (8) hemorrhage, (9) metastasis, (10) musculoskeletal findings (e.g., pathologic fracture), (11) intracranial findings (e.g., infarct, encephalitis), (12) catheter- or device-related problems, (13) inflammation or infection elsewhere (e.g., not related to bowel, urinary tract, or lungs), and (14) others. Metastases were categorized into “new or increased”, “decreased”, or “unchanged”.

The following clinical information was extracted from our electronic medical records database: age, gender, type of primary tumor, ethnicity, chief complaint, and disposition after the visit to the ER (admission vs. discharge), and survival. Chief complaints were categorized as fever, chest symptoms (e.g., chest pain, cough, shortness of breath), abdominal pain, gastrointestinal symptoms (e.g., nausea, vomiting, diarrhea), genitourinary symptoms (e.g., dysuria, hematuria, scrotal swelling), back pain or discomfort, Neurological symptoms (e.g., altered mental status, dizziness, weakness, headache, seizures), extremity complaints (e.g., extremity pain or swelling), lymphadenopathy, wound or treatment-related issues, trauma, catheter- or device-related problems (e.g., pulled out tube, central venous catheter malfunction), laboratory test abnormality (e.g., elevated liver function tests, elevated creatinine), and suspected progression of disease [4]. More than one chief complaint could be attributed to the patient’s visit based on review of the clinical notes.

### Image acquisition

Imaging studies were requested at the discretion of the referring physicians with the anatomical coverage and acquisition protocol modified by radiologists, with the aim of tailoring imaging to the patient’s chief complaint on presentation to the ER. For example, chest radiographs with postero-anterior and lateral view were performed when pneumonia was suspected. Abdominal radiographs were initially performed for surveillance of patients with abdominal pain, but additional imaging studies such as right upper quadrant US or pelvic US were done in accordance with localization of the symptoms and corresponding differential diagnoses [8]. CT or MRI was performed when there was an indication to assess the tumor (primary or metastatic) extent or when further information that could not be assessed by radiographs or US was needed, such as complications related to pneumonia, pulmonary embolism, intra-abdominal abscess, or stroke with protocols tailored to the clinical question (e.g., CT chest with or without contrast, CT chest with pulmonary angiography, CT abdomen and pelvis with contrast, and MRI brain with diffusion and contrast) [4, 9–11].

### Statistical analysis

Continuous variables are summarized as median and interquartile ranges (IQR); and categorical variables are described as frequencies and percentages. We assessed the relationship between ER imaging positivity and clinical variables (i.e., age, type of primary tumor, chief complaint and disposition) using the chi-square test, Fisher’s exact, or Wilcoxon rank-sum test as appropriate. Baseline demographics were summarized at the patient level and analyses of ER imaging findings and clinical variables were done at the level of each visit. If there were multiple imaging studies performed on a single visit related to multiple chief complaints, we representatively used the more advanced and definitive modality that answered the clinical question of that visit. For example, if a right upper quadrant US identified acute cholecystitis after an inconclusive abdominal radiograph, US was chosen. If a chest CT identified increased pulmonary metastases after an inconclusive chest radiograph, the CT was selected as the representative imaging modality for that visit. OS was determined by Kaplan-Meier method following the last ER visit for each patient and tested statistical significance using the log-rank test. Univariate and multivariate Cox-proportional hazards regression model was used to determine variables significantly associated with overall survival in terms of hazard ratios (HR). Variables that were significant on univariate analysis were considered for candidates in the multivariate model. Statistical software R (version 3.6.1; R Foundation for Statistical Computing, Vienna, Austria) as used for analysis with *P* values < 0.05 considered to be statistically significant.

## Results

### Baseline demographics

Patient demographics are summarized in Table 1. There were 135 (51.7 %) males and 126 (48.3 %) females with a median age of 11 years (IQR 5–16 years). The most commonly identified ethnicities were white (141 [54.0 %]) and Hispanic (36 [13.8 %]). The most common types of primary tumors were bone and soft tissue tumors (72 [27.6 %] most commonly osteosarcoma [*n* = 25], alveolar rhabdomyosarcoma [*n* = 9], and Ewing sarcoma [*n* = 7]), hematologic malignancies (68 [26.1 %]), neuroblastoma (51 [19.5 %]), and central nervous system (CNS) tumors (53 [15.2 %]). The median number of visits to the ER was 1 (range 1–8; IQR 1–1). Ninety (34.5 %) patients died during a median follow-up of 590 days (IQR 157–1,127 days) from their last ER visit.

**Table 1:** Baseline demographics of 261 pediatrics cancer patients who visited the emergency room

Characteristic	*N* = 261
Age ^a^	11 (5, 16)
Gender	
Male	135 (51.7%)
Female	126 (48.3%)
Primary cancer type	
Bone and soft tissue tumors	72 (27.6%)
Hematologic malignancies	68 (26.1%)
Neuroblastoma	51 (19.5%)
Central nervous system tumors	34 (13.0%)
Retinoblastoma	12 (4.6%)
Other tumors	24 (9.2%)
Ethnicity	
White	141 (54.0%)
Hispanic	36 (13.8%)
Black	29 (11.1%)
Asian	26 (10.0%)
Others/unknown	29 (11.1%)
No. of visits	
1	206 (78.9%)
2	35 (13.4%)
3	14 (5.4%)
4	3 (1.1%)
5	2 (0.8%)
8	1 (0.4%)

Unless otherwise indicated, data are no. of patients with percentage in parenthesis

^a^ Data are median with interquartile range in parentheses

### Characteristics of ER visits

Patients most commonly visited the ER due to the following chief complaints: chest symptoms (100 [28.7 %]), fever (99 [28.4 %]), neurological symptoms (64 [18.4 %]), and gastrointestinal symptoms (63 [18.1 %]). After the ER visit, 196 (56.3 %) patients were admitted. The details of the clinical variables stratified to ER imaging positivity are shown in Table 2. ER imaging was more frequently positive when progression of disease was suspected (*p* = 0.01). There were no significant differences in the positivity of ER imaging with regards to the other reasons for the ER visit (*p* = 0.05–0.94).

**Table 2:** Characteristics of visits stratified to emergency room imaging positivity

Characteristic	All visits (*N* = 348)	ER imaging negative (*N* = 156)	ER imaging positive (*N* = 192)	*p*-value
Age ^a^	11 (6, 15)	11 (6, 15)	10 (6, 15)	0.82^†^
Gender				0.83
Male	184 (52.9%)	84 (53.8%)	100 (52.1%)	
Female	164 (47.1%)	72 (46.2%)	92 (47.9%)	
Type of primary cancer				0.92
Bone & soft tissue tumors	87 (25.0%)	41 (26.3%)	46 (24.0%)	
Hematologic malignancies	95 (27.3%)	45 (28.8%)	50 (26.0%)	
Neuroblastoma	62 (17.8%)	28 (17.9%)	34 (17.7%)	
Central nervous system tumors	53 (15.2%)	22 (14.1%)	31 (16.1%)	
Retinoblastoma	14 (4.0%)	5 (3.2%)	9 (4.7%)	
Other tumors	37 (10.6%)	15 (9.6%)	22 (11.5%)	
Ethnicity				0.28
White	186 (53.4%)	83 (53.2%)	103 (53.6%)	
Hispanic	49 (14.1%)	18 (11.5%)	31 (16.1%)	
Black	39 (11.2%)	21 (13.5%)	18 (9.4%)	
Asian	32 (9.2%)	18 (11.5%)	14 (7.3%)	
Oher or unknown	42 (12.1%)	16 (10.3%)	26 (13.5%)	
Admission	196 (56.3%)	63 (40.4%)	133 (69.3%)	<0.01
Reason for visit				
Chest symptoms	100 (28.7%)	43 (27.6%)	57 (29.7%)	0.75
Fever	99 (28.4%)	48 (30.8%)	51 (26.6%)	0.46
Neurological symptoms	64 (18.4%)	24 (15.4%)	40 (20.8%)	0.24
GI symptoms	63 (18.1%)	29 (18.6%)	34 (17.7%)	0.94
Abdominal pain	57 (16.4%)	23 (14.7%)	34 (17.7%)	0.55
Laboratory test abnormality	25 (7.2%)	9 (5.8%)	16 (8.3%)	0.48
Extremity complaints	18 (5.2%)	7 (4.5%)	11 (5.7%)	0.78
Evaluation of disease	13 (3.7%)	1 (0.6%)	12 (6.2%)	0.01^‡^
Device	13 (3.7%)	8 (5.1%)	5 (2.6%)	0.34
Wound or treatment-related	12 (3.4%)	7 (4.5%)	5 (2.6%)	0.51
Trauma	10 (2.9%)	4 (2.6%)	6 (3.1%)	>0.99
Genitourinary symptoms	9 (2.6%)	1 (0.6%)	8 (4.2%)	0.05^‡^
Back pain/discomfort	7 (2.0%)	2 (1.3%)	5 (2.6%)	0.47^‡^
Lymphadenopathy	2 (0.6%)	0 (0.0%)	2 (1.0%)	0.5^‡^
Modality				<0.01
Radiograph	195 (56.0%)	115 (73.7%)	80 (41.7%)	
Computed tomography	88 (25.3%)	22 (14.1%)	66 (34.4%)	
Magnetic resonance imaging	40 (11.5%)	12 (7.7%)	28 (14.6%)	
Ultrasound	21 (6.0%)	7 (4.5%)	14 (7.3%)	
Nuclear Medicine	4 (1.1%)	0 (0.0%)	4 (2.1%)	

Unless otherwise indicated, data are no. of visits with percentage in parenthesis

Unless otherwise indicated, chi-square test was used for statistical analysis

^a^ Data are median with interquartile range in parentheses

*ER* emergency room

^†^ Wilcoxon rank-sum test

^‡^Fisher's exact test

### ER imaging characteristics

**Fig. 2:**
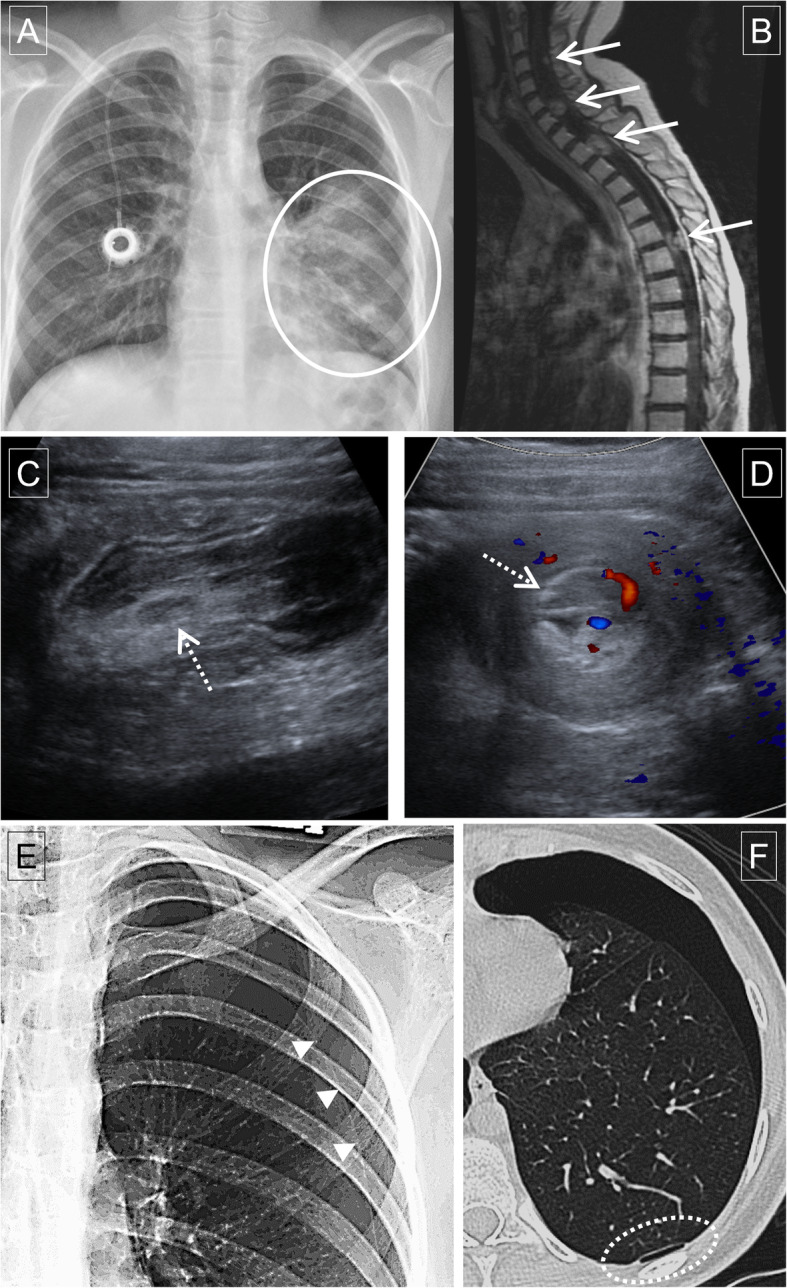
Representative cases of ER imaging findings in pediatric cancer patients. **a** 14-yo male with chronic myelogenous leukemia comes to the ER with fever and cough. Chest radiograph showed consolidation (circle) in the left lung consistent with pneumonia. Patient was alive at 838 days after the visit. **b** 15-year-old female with undifferentiated soft tissue sarcoma of right skull base presents with weakness and urinary retention. MRI spine showed increased leptomeningeal and intramedullary metastases (arrows) on sagittal contrast-enhanced T1-weighted image causing cord compression shown on axial T2-weighted image. Patient died 132 days after the visit. **c**-**d** 10-year-old female with post-transplant lymphoproliferative disorder after a remote cardiac transplantation presenting with abdominal pain, nausea, and vomiting. Abdominal ultrasound (**c**) and Doppler studies (**d**) demonstrate long segmental small bowel intussusception in the left upper quadrant with a mass suspicious lead-point (broken arrows) which was confirmed after surgery. Patient was alive at 1543 days after the visit. **e**-**f** 14-year-old female with B-lymphoblastic lymphoma presents with chest pain. Chest radiograph (**e**) showed pneumothorax (pleural line shown with arrowheads). Further evaluation with CT (**f**) identified numerous left subpleural blebs (broken circle) that was speculated to be causative of the pneumothorax. Patient was alive at 1562 days after the visit

The distribution and frequency of ER imaging was as follows: radiograph (195 [56.0 %]), CT (88 [25.3 %]), and MRI (40 [11.5 %]) (Table 2). The breakdown of ER imaging findings is shown in Table 3 and details of modality and their coverage is summarized in Table 4. Most common imaging findings were increased metastases (50 [14.4 %]), pneumonia (47 [13.5 %]), lung problems other than pneumonia (12 [3.4 %]), and infectious/inflammatory processes in other sites (10 [2.9 %]). Representative cases of these common findings are provided in Fig. 2. ER imaging was positive in 192 (55.2 %) visits. The prevalence of positive ER imaging was significantly greater in patients that were admitted compared to those that were discharged (69.3 % [133/192] vs. 40.4 % [63/156], *p* < 0.01).

**Table 3:** Spectrum and frequency of emergency room imaging findings at 348 visits

Characteristic	*N* = 348
Positive	192 (55.2%)
Metastasis or primary tumor	69 (19.8%)
*Increased*	*50 (14.4%)*
*Unchanged*	*10 (2.9%)*
*No comparison*	*8 (2.3%)*
*Decreased*	*1 (0.3%)*
Pneumonia	47 (13.5%)
Other lung findings	12 (3.4%)
Inflammation/infection (others)^a^	10 (2.9%)
Bowel obstruction	8 (2.3%)
Musculoskeletal	8 (2.3%)
Bowel inflammation/infection	6 (1.7%)
Abscess or complicated fluid	6 (1.7%)
Bleeding/hematoma	4 (1.1%)
Catheter- or device-related problem	6 (1.7%)
Genitourinary inflammation/infection	6 (1.7%)
Pneumatosis	4 (1.1%)
Brain abnormalities	2 (0.6%)
Other	4 (1.1%)
Negative	156 (44.8%)

Data are no. of visits with percentage in parenthesis

^a^ Infection in body parts other than the bowel, urinary tract, and lungs

**Table 4:** Breakdown of emergency room imaging by modality and coverage

Characteristic	All visits (*N* = 348)	ER imaging negative (*N* = 156)	ER imaging positive (*N* = 192)
Chest Radiograph	134 (38.5%)	70 (44.9%)	64 (33.3%)
Abdomen Radiograph	50 (14.4%)	30 (19.2%)	20 (10.4%)
Brain CT	38 (10.9%)	17 (10.9%)	21 (10.9%)
Brain MR	23 (6.6%)	9 (5.8%)	14 (7.3%)
Musculoskeletal Radiograph	23 (6.6%)	13 (8.3%)	10 (5.2%)
Abdomen CT	22 (6.3%)	3 (1.9%)	19 (9.9%)
Abdomen US	12 (3.4%)	3 (1.9%)	9 (4.7%)
Chest CT	7 (2.0%)	0 (0.0%)	7 (3.6%)
Spine MR	5 (1.4%)	0 (0.0%)	5 (2.6%)
Chest and abdomen CT	4 (1.1%)	1 (0.6%)	3 (1.6%)
Head and neck MR	4 (1.1%)	1 (0.6%)	3 (1.6%)
Musculoskeletal CT	4 (1.1%)	1 (0.6%)	3 (1.6%)
Vascular US	4 (1.1%)	3 (1.9%)	1 (0.5%)
Abdomen MR	3 (0.9%)	0 (0.0%)	3 (1.6%)
Head and neck CT	3 (0.9%)	0 (0.0%)	3 (1.6%)
Other body part US	3 (0.9%)	0 (0.0%)	3 (1.6%)
Spine Radiograph	2 (0.6%)	1 (0.6%)	1 (0.5%)
Nuclear medicine	2 (0.6%)	0 (0.0%)	2 (1.0%)
Other body part Radiograph	2 (0.6%)	2 (1.3%)	0 (0.0%)
Musculoskeletal MR	1 (0.3%)	1 (0.6%)	0 (0.0%)
Spine CT	1 (0.3%)	1 (0.6%)	0 (0.0%)
Chest MR	1 (0.3%)	0 (0.0%)	1 (0.5%)

Data are no. of visits with percentage in parenthesis

*CT* computed tomography, *MRI* magnetic resonance imaging, *US* ultrasound

### Factors associated with survival

**Fig. 3:**
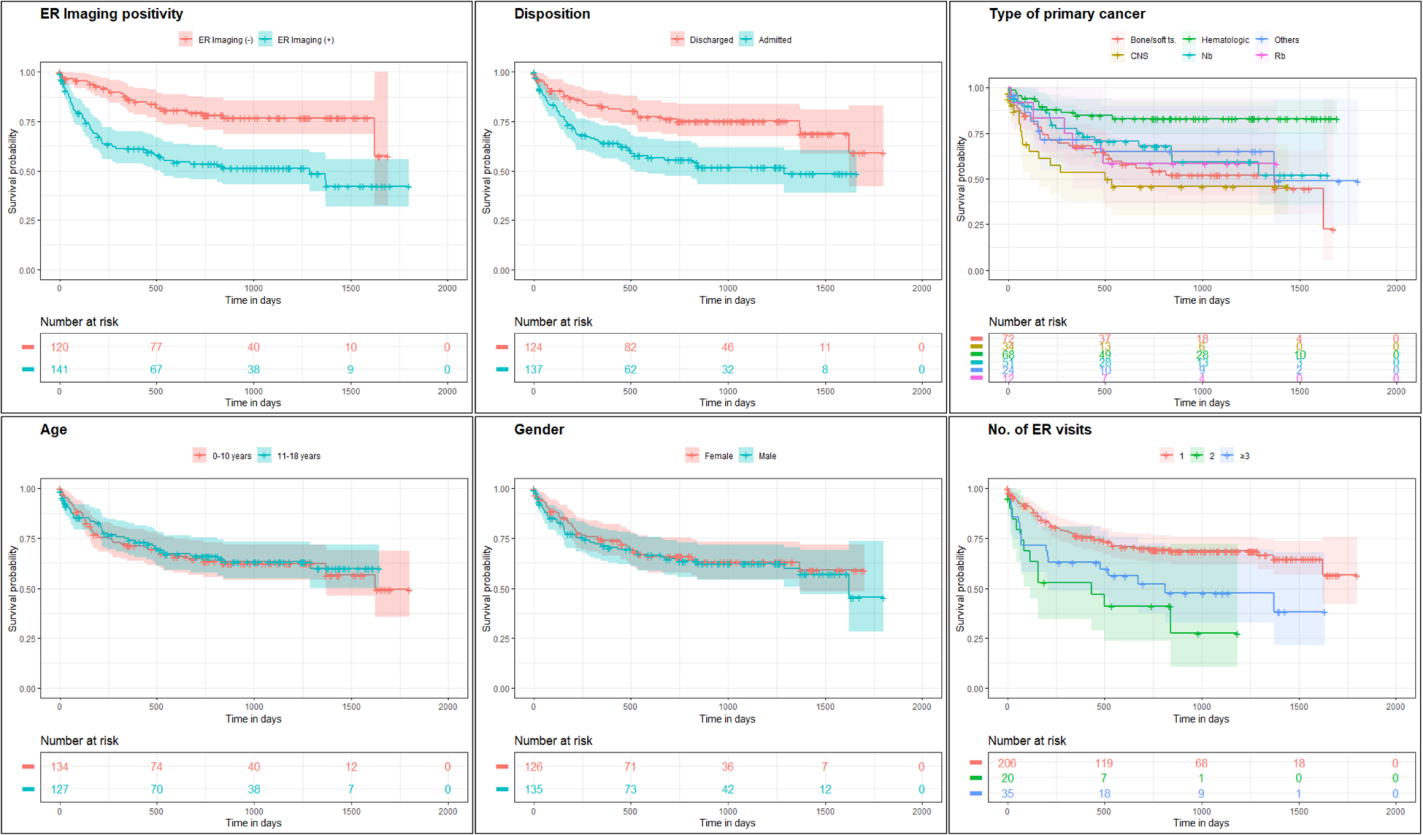
Kaplan-Meier survival curves stratified to ER imaging positivity and clinical variables. **a** ER imaging positivity; **b** admission; **c** primary cancer type; **d** age; **e** gender; **f** number of ER visits

Survival curves stratified ER imaging positivity and clinical variables are shown in Fig. 3 and their univariate and multivariate HRs are provided in Table 5. At univariate analysis, positive ER imaging, disposition (admission vs. discharge), primary cancer type and number of ER visits were significantly associated with survival (*p* < 0.01). At multivariate analysis, factors independently associated with survival were: positive ER imaging (HR = 2.35 [95 % CI 1.44–3.83, *p* < 0.01), admission (HR = 1.86 [95 % CI 1.17–3.00], *p* < 0.01), and multiple ER visits (HR = 1.98 [95 % CI 1.15–3.41], *p* = 0.01 for 2 visits and HR = 3.08 [95 % CI 1.62–5.83], *p* < 0.01 for 3 or more visits compared with one) were associated with worse survival; whereas patients with hematologic malignancies were associated with better survival (HR = 0.25 [95 % CI 0.13–0.50], *p* < 0.01).

**Table 5:** Hazard ratios of multiple clinical variables on uni- and multivariate survival analysis

Variable	Stratification	Univariate HR (95% CI)	*p*-value	Multivariate HR (95% CI)	*p*-value
ER imaging	Positive	2.84 (1.78–4.53)	<0.01	2.35 (1.44–3.83)	<0.01
	Negative	1	reference	1	reference
Admission	Admitted	2.12 (1.34–3.30)	<0.01	1.86 (1.16–2.97)	<0.01
	Discharged	1	reference	1	reference
Cancer type	CNS tumors	1.32 (0.72–2.43)	0.38	1.45 (0.78–2.70)	0.24
	Hematologic malignancies	0.28 (0.14–0.56)	<0.01	0.25 (0.13–0.50)	<0.01
	Neuroblastoma	0.72 (0.41–1.28)	0.26	0.62 (0.35–1.11)	0.11
	Retinoblastoma	0.82 (0.32–2.11)	0.69	0.90 (0.35–2.33)	0.83
	Others	0.77 (0.36–1.67)	0.51	0.64 (0.29–1.44)	0.28
	Bone & soft tissue tumors	1	reference*	1	reference*
Gender	Male	1.10 (0.73–1.66)	0.66		
	Female	1	reference		
Age (years)	11-18	0.95 (0.62–1.43)	0.79		
	0-10	1	reference		
No. ER visits	≥3	2.96 (1.59–5.52)	<0.01	3.08 (1.62-5.83)	<0.01
	2	1.97 (1.16–3.34)		1.98 (1.15–3.41)	0.01
	1	1	reference	1	reference

*CI* confidence interval, *CNS* central nervous system, *ER* emergency room, *HR* hazard ratio

*Log-rank *p*-value <0.01 for analysis across all cancer types

## Discussion

In the current study, we assessed the spectrum and frequency of imaging modalities and findings in pediatric cancer patients visiting the ER at a tertiary cancer center. ER imaging was positive in just over half of the visits (56 %) with a large proportion of them being attributed to increased burden of either metastatic disease or the primary tumor and abnormalities in the chest (e.g., pneumonia and other findings). Based on these findings, we suggest that ER imaging can often identify the underlying structural cause for the reason of the ER visit in pediatric patients with cancer. Helping explain the root cause allows the ER physician to provide the best possible care including timely diagnosis of the problem, optimal triage and allocation of the child to the appropriate specialty, and potentially shortening the time to definitive intervention. In line with this, positive ER imaging was associated with a greater rate of admission in the current study (*p* < 0.01).

Pediatric patients with cancer visited the ER for a wide range of reasons, among which chest symptoms, fever, neurological symptoms and gastrointestinal symptoms were common. ER imaging commonly revealed pneumonia, other lung problems, and infectious/inflammatory processes in other sites which are expected to occur related to not only the various types of treatments directed at cancer (e.g., surgery, chemotherapy, and radiation treatment), but also manifestations of the cancer itself [12–14]. Neurological symptoms were relatively common, probably at least partly related to the fact that a sizable proportion of patients had CNS tumors (13 %). Regarding positive ER imaging findings, the most common was increased metastases (14.4 %) even as suspected disease progression was one of the main reasons for the visit in only a minority of the visits (3.7 %). This has important clinical implications in that not only progression of disease should be kept in mind as one of the top differentials when confronting a pediatric patient with cancer visiting the ER, they may manifest with a myriad of different symptoms and signs not necessarily seemingly related to progression.

Positive ER imaging and inpatient admission were significantly and independently associated with worse survival in this cohort of pediatric patients with cancer (HR = 2.35 and 1.86, respectively, *p* < 0.01 for both). Identification of positive findings on ER could be associated with survival for several reasons. First, there was a significant relationship between ER imaging status and disposition – that is, patients with positive ER imaging were more likely to be admitted during their ER visit (69.3 % vs. 40.4 %, *p* < 0.01). This suggests that having positive ER imaging generally represents a more serious condition, either it be greater metastatic burden or complications related to treatment [15–17]. Additionally, the number of ER visits were significantly associated with survival demonstrating a dose-response relationship (HR = 3.08 for 3 or more visits; HR = 1.98 for 2 visits; *p* ≤ 0.01). Furthermore, the correlation may have been strengthened by the fact that patients with hematologic malignancies fare better in terms of OS and are less likely to have positive imaging as their disease progression is often not readily apparent on imaging when compared with patients with solid tumors. Although not all ER visits may necessarily be life-threatening or result in shorter survival, increased number of visits may partly reflect the patients’ status of worsening tumor burden (that may manifest in many ways) despite treatment or other conditions related to treatment or their sequelae. In addition, type of primary cancer was also related with survival. Hematologic malignancies, inclusive of leukemias and lymphomas demonstrated better survival than other types of cancers (HR = 0.25, *p* < 0.01). This may reflect the fact that pediatric patients with these cancers are known to show favorable clinical outcomes and have benefitted from marked progress via clinical trials with substantially steeper decline in overall mortality rates over the last 3 decades compared with other tumors (e.g., solid and CNS tumors) [18, 19].

There were some limitations in this study. First, the methodology comprised a retrospective analysis performed in a single tertiary cancer center which could result in inherent bias. Other institutions, either those that are not cancer-dedicated, non-tertiary or treat different subtypes of pediatric cancers, may demonstrate more diversity in the patient population in terms of volume and spectrum of pediatric cancer patients. Specifically, our data cannot be extrapolated to the non-cancer patient. Second, we comprehensively assessed all types of imaging modalities performed at the ER visit. Therefore, the ER imaging positivity and their relationship with outcomes (e.g., disposition and survival) may depend on the indications for each type of imaging modality among different centers. Third, although we identified differences in survival between subgroups of patients with different primary cancers, we could not look into more detail regarding each type of cancer due to paucity of patients with certain types of tumors, especially those arising from the viscera (e.g., lung, liver, pancreas, kidney, and colon). Nevertheless, this was not the primary aim of this study.

## Conclusions

When imaging was performed, the reason for the ER visit was identified in more than half of pediatric cancer patients. Positive ER imaging was associated with increased need for admission and worse survival.

## Data Availability

Individual patient data is only accessed by authors who received approval of institutional review board according to federal laws and regulations and institutional/departmental policies. Aggregate summary data may be provided upon request in keeping with protection of healthcare information.

## Abbreviations

CT: Computed tomography
ER: Emergency room
IQR: Interquartile range
MRI: Magnetic resonance imaging
PET: Positron emission tomography
US: Ultrasound
